# The impact of direct health facility financing on MNCH service provision: results from a comparative, before-after study in Pwani Region, Tanzania

**DOI:** 10.1186/s12913-024-11917-w

**Published:** 2024-11-18

**Authors:** Kyoung Kyun Oh, Joy G. Ferdinand, Ntuli A. Kapologwe, Benedicto M. Ngaiza, Joyce M. Gordon, Doowon Lim, Alfred E. Ngowi, Swabaha A. Yusuph, Hayoung Kim, Hansol Park, Sooyoung Ahn, Bok Hyun Nam, Chang-yup Kim

**Affiliations:** 1 Korea Foundation for International Healthcare (KOFIH) Tanzania Office, Dar es Salaam, Tanzania; 2 School of Health & Wellbeing, University of Glasgow, Glasgow, UK; 3 Ministry of Health, Dodoma, Tanzania; 4 Regional Secretariat- Pwani Region, Regional Commissioner’s Office, Pwani, Tanzania; 5 Han-a Urban Research Institute, Seoul, Korea; 6 Korea Foundation for International Healthcare (KOFIH), Seoul, Korea; 7 Graduate School of Public Health, Seoul National University, Seoul, Korea

**Keywords:** Health System Strengthening, Health Financing, MNCH, DHFF, DHIS2, CEmONC, ODA, KOFIH, Tanzania

## Abstract

**Background:**

Pwani Regional Secretariat in Tanzania implemented the Maternal, Neonatal, and Child Health Project (2016–2022) through Direct Health Facility Financing (DHFF), which allocates funds directly to health facilities. This study assessed the impact of the six-year DHFF project in Pwani region.

**Methods:**

The study utilised District Health Information Software 2 data from 18 intervention health facilities in Pwani region. Control groups comprised an equal number of facilities from Pwani and Dodoma regions where the project was not implemented. Key indicators assessed included ‘ANC 4 + Rate (%)’, ‘Percentage of Mothers tested for Anaemia during ANC’, ‘Caesarean Section Delivery Rate (%)’, ‘Percentage of Mothers and Newborns receiving PNC services within 48 hours’, ‘Delivery Complication Rate (%)’, and ‘SBA Delivery Rate (%)’ which are associated with the project interventions. The impact of the project was analysed using a paired sample t-test comparing baseline and endline data. We evaluated the significance of the dependent variables using one-way ANOVA with control groups, with the Tukey-Kramer test for post hoc analysis. Chi-square test assessed the significance of Caesarean Section Delivery Rate and the relationship between variables and health facility conditions. Pearson correlation test was used for significance between funding size and the change of MNCH variables. Statistical significance at 0.05 was calculated.

**Results:**

The project showed limited positive impacts, only in the ‘Percentage of Mothers tested for Anaemia during ANC’ (****p < 0.0001), ‘Percentage of Newborns receiving PNC within 48 hours’ (**p = 0.0095), and ‘SBA Delivery Rate’ (***p = 0.0043). The health facility assessment identified positively influencing factors on service delivery, such as facility type (*p = 0.0347), distance to the facility (****p < 0.0001), and internet connectivity (*p = 0.0186). We found that the project did not improve most MNCH indicators, including the CEmONC coverage (χ2 = 2.82, *p* = 0.2448, df = 2), which was known to be the leading outcome.

**Conclusion:**

The project had limited impacts on MNCH outcomes due to various factors. While the health facility assessment highlighted positive influences on service delivery, significant areas for improvement remain, including referral systems and infrastructure. Operational research findings indicate that the effectiveness of the DHFF could be enhanced by refining its management and governance structures.

## Background

While Tanzania sustained one of the highest Maternal, Neonatal, and Child Health (MNCH) burdens in the world over the past decades, it has made notable progress in reducing neonatal mortality (NM), infant mortality rate (IMR), under-5 mortality rate (U5M), and maternal mortality ratio (MMR). In 2015, NM, IMR, U5M, and MMR were recorded at 25, 43, and 67 per 1,000 live births, and 556 per 100,000 live births [1]. By 2022, these figures had declined to 24, 33, and 43 per 1,000 live births, and 220 per 100,000 live births, respectively [1–3].

Pwani region in the Coastal Zone of Tanzania plays a leading role to the country’s progress in achieving MNCH goals. Among the 30 regions in the United Republic of Tanzania (URT), Pwani region is recognised for having comparatively stronger health indicators particularly in MNCH outcomes such as child vaccination, fertility rate, benefitting from a well-distributed mix of rural and urban environments [3, 4]. Although MNCH indicators in the region are relatively positive, largely due to improved access to health services [3, 4], the region continues to face considerable challenges in achieving the Sustainable Development Goal 3 (SDG 3) target. Particularly, key MNCH concerns, including inadequate antenatal care (ANC), postpartum haemorrhage, pregnancy-related infections, obstructed labour, complications from abortion, delayed postnatal care (PNC) for both mothers and newborns, competency of health care providers and poor access to MNCH services, remain critical contributors to NM, IMR, U5M and MMR [3, 5, 6]. These factors, coupled with other direct and indirect MNCH complications, continue to impose a significant public health burden on the region [3, 5, 6].

**Fig. 1:**
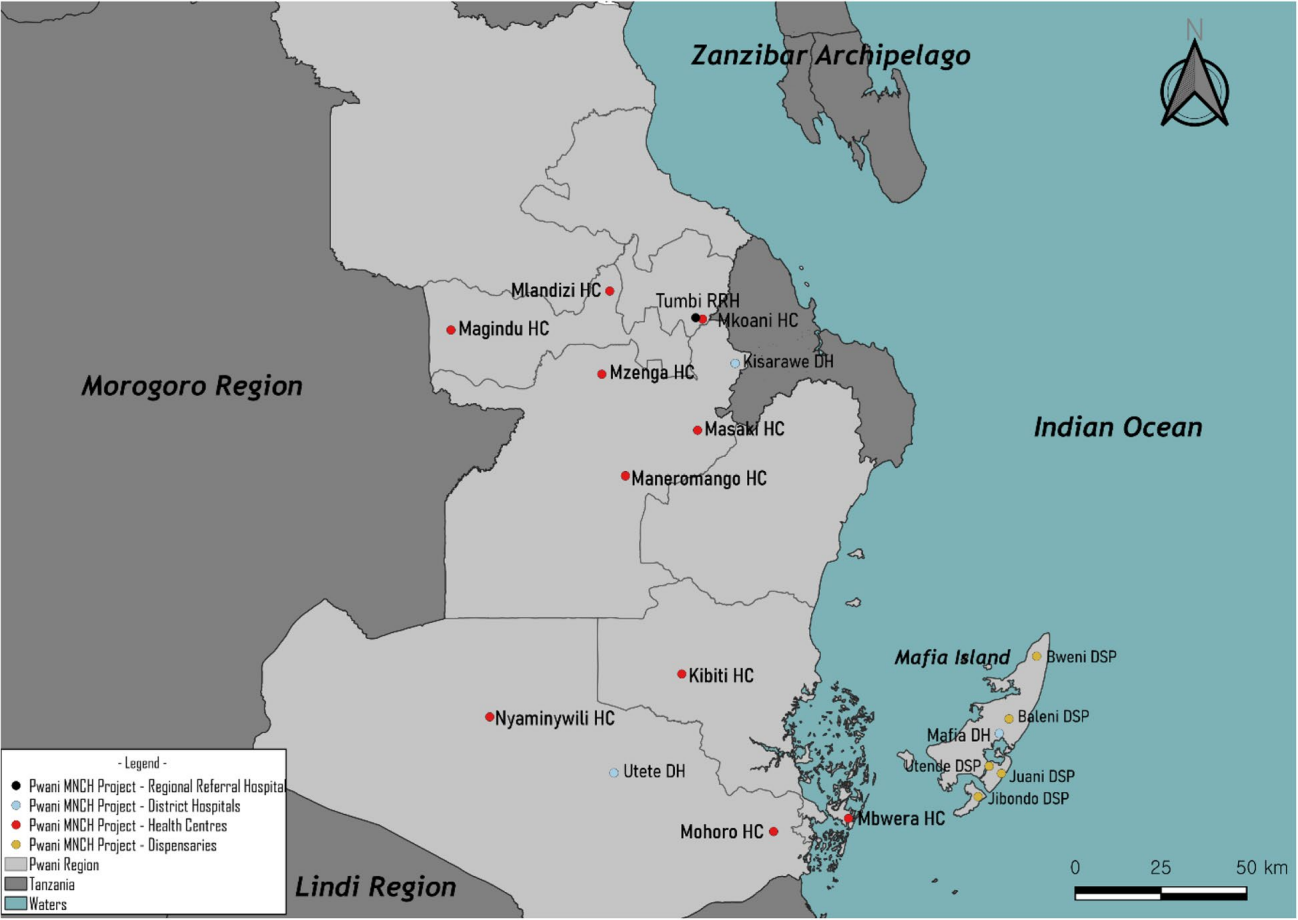
A site map of the Health System Strengthening Project focused on MNCH through the Direct Health Facility Financing system in Pwani region

Quality MNCH care in sufficient budget plans with appropriate service provision plays a vital role in preventing stillbirths, maternal and newborn deaths [7]. Health financing is a critical component of health systems aiming to sustain quality health services and health service delivery [8]. The Government of Tanzania (GoT) has made significant progress in strengthening health financing [9], one of the footmarks is an introduction of the Direct Health Facility Financing (DHFF) launched in the 2017/2018 fiscal year across Tanzania. The DHFF is defined as a direct provision of government or external funds to the health facility to fulfil operational requirements [9–11]. The facility prepares the budget plan, and the execution is carried out by the facility through direct health financing from sources. The DHFF aims to improve health system performance through disbursing funds to the primary health care (PHC) facilities to facilitate health service delivery through a lens of achieving Universal Health Coverage (UHC). Pwani Regional Secretariat, in collaboration with the Ministry of Health (MOH) and the President’s Office, Regional Administration and Local Government (PO-RALG), adopted DHFF, funded by Korea Foundation for International Healthcare (KOFIH) in order to fill the gaps in health financing and MNCH issues. This six-year project, launched from 2016 to 2022, focused on MNCH at 19 health facilities (1 Regional Referral Hospital, 3 District Hospitals, 10 Health Centres and 5 Dispensaries), aimed at mainly strengthening Comprehensive Emergency Obstetric and Newborn Care (CEmONC) aligning with national strategy [12] (Fig. 1).

This project was to improve more equitable MNCH accessibility to the community people through increasing health facility-based delivery, ANC and PNC, a safe birth environment with skilled birth attendances (SBAs), and scaling up the CEmONC services [12–15]. To achieve it, the project provided some intervention points such as (1) Decentralising CEmONC services, including construction and refurbishment, (2) Strengthening referral systems, (3) Mentorship programme, and (4) Strengthening leadership and management. Simultaneously, the external funder directly provides resources to health facilities through GoT’s health financing systems to expedite operational requirements. The significant arms of intervention were health infrastructure support, CEmONC and BEmONC service delivery and relevant training for capacity building of health care providers.

Although the DHFF has played a pivotal role in decentralising health systems in Tanzania, there remain critical gaps in understanding its influence on health systems, particularly regarding the delivery of MNCH services from a vertical perspective. We analysed the results of the project outcomes and assessed the impacts of the DHFF on MNCH in Pwani region based on the key MNCH variables collected from the District Health Information Software 2 (DHIS2) covering the project period from 2016 (baseline) to 2022 (endline) to derive actionable lessons for future implementation [12–14, 16].

## Methods

### Study settings

Tanzania’s health system operates in a decentralised, pyramid structure [17]. Primary health care facilities include dispensaries (DSPs), health centres (HCs), and district hospitals (DHs), are all under the supervision of the PO-RALG. Secondary and tertiary health care facilities - Regional Referral Hospitals (RRHs), Zonal Hospitals (ZHs), Specialised Hospitals (SHs), and National Hospitals (NHs) - are under the governance of the MOH [17]. Both the MOH and the PO-RALG are responsible for providing guidelines, training, and oversight. Pwani region has an estimated area of 32,407 km^2^ and a population of 2,024,947 (m: 998,616 / f: 1,026,331) [4]. The total population of childbearing women is 51.3%, and the urban and rural dweller rate is 41.3% and 58.7%, respectively [4]. There are 542,919 households (mean HH size: 3.7), 108 wards and 9 councils [4]. Among the 9 councils, 6 councils (Kibaha District, Kibaha Town, Kisarawe District, Rufiji District, Mafia District and Kibiti District) were included in the project. The Council Health Management Team (CHMT) is responsible for health issues at the council level. The total population of these areas account for 47.9% (969,677 (m: 481,219, f: 488,458)) of the whole population in Pwani region [4].

As control groups, the same number with similar characteristics of health facilities were selected from two regions; Pwani and Dodoma (18 facilities, respectively). From Pwani region, all health facilities, except for DSPs, were chosen in Bagamoyo, Chalinze, and Mkuranga Councils, where the project was not implemented. From Dodoma region, health facilities except one DH (from Kondoa Council) were selected from Kongwa and Mpwapwa Councils. To reduce bias, we purposively selected regions and then randomly screened health facilities by such criteria: (1) Non-project area, (2) Public facility, (3) Similar number of ANC as the intervention group, (4) Catchment population, and (5) Data availability. The Health Facility Registry System (www.hfrs.moh.go.tz) was used for screening criteria information.

### Theory of change

**Fig. 2:**
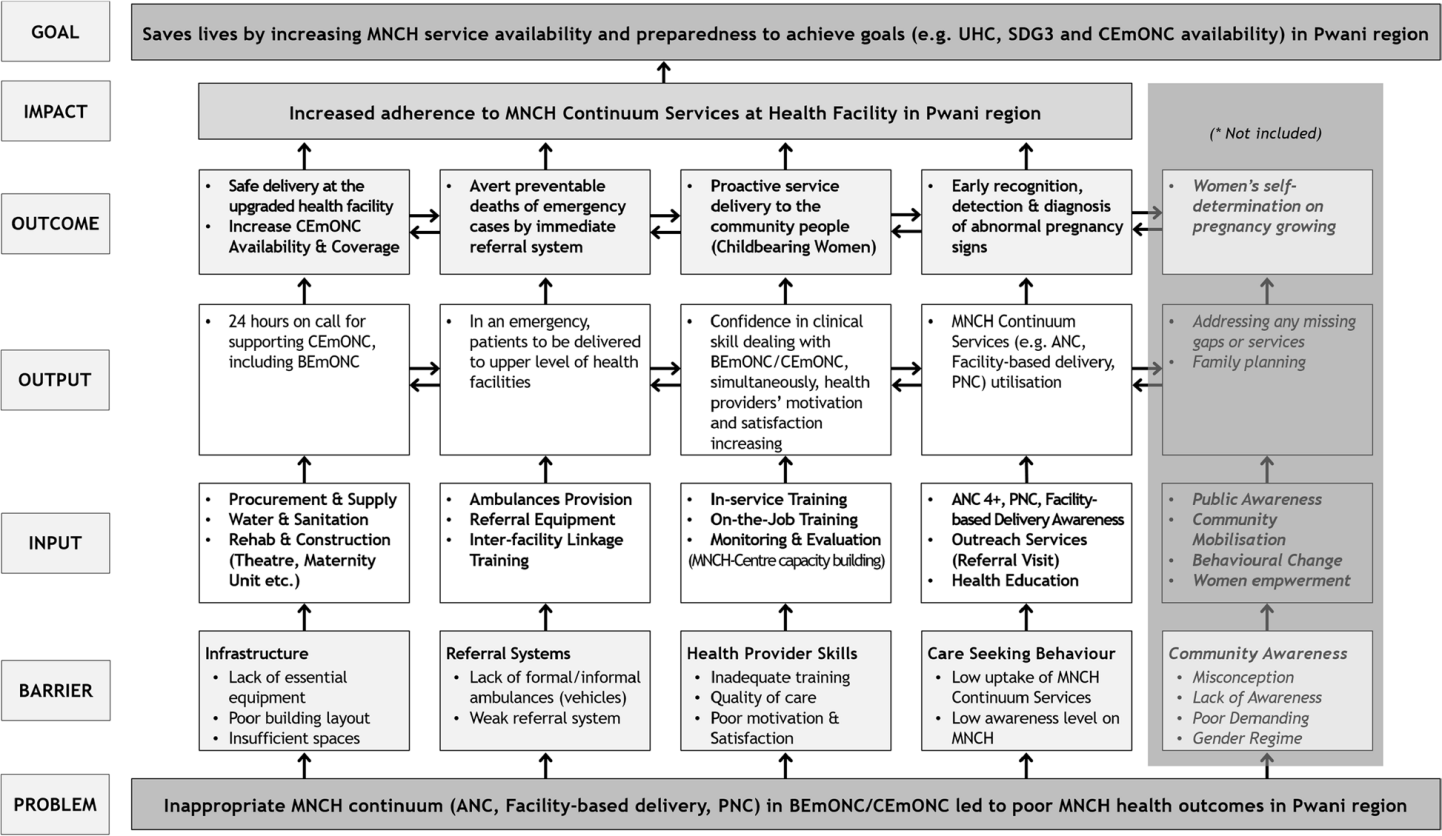
Theory of Change on Health System Strengthening Project focused on MNCH through the Direct Health Facility Financing

A theory of change (ToC) for health facility improvement via the DHFF outlines pathways linking the DHFF impacts to MNCH outcomes. It identifies health system bottlenecks such as financing, human resources, service delivery, health information, supply chain and governance [17, 18]. In this case, increased quality of maternal health care and improved health facilities are related to achieving CEmONC at the PHC facilities to reduce maternal and neonatal mortality. The authors refined the ToC for prospective evaluations based on the project design matrix (Fig. 2). We illustrated the key 5 pillars such as facility infrastructure, referral systems, health providers skills, care-seeking behaviour, and community awareness to describe increasing MNCH continuum services at the PHC level in Pwani region. In turn, the impact aims to save lives by increasing MNCH service availability and preparedness. Given that the project was primarily focused on the health service provider level, community-level intervention components such as community awareness were excluded from the ToC.

### Project interventions

#### Decentralising CEmONC services

To enable HCs to provide CEmONC services, the project upgraded 7 HCs where Caesarean Section Delivery was not available prior to project initiation. Operating theatres, maternity wards, and laboratories were built or refurbished. Infrastructure such as water and sanitation (WASH) systems and infectious disease prevention and control (IDPC) were upgraded. Health centres were also provided with essential medical equipment. There were 3 DHs referred from HCs received various infrastructure improvements and provision of essential medical equipment. Between 2016 and 2022, 374 health care providers, including clinicians and nurses, were trained to deliver surgical services (obstetric surgery, theatre management, and anaesthesia) in upgraded HCs and supported DHs. In addition, 307 health care providers based in maternity wards were trained to deliver MNCH services with high quality.

#### Strengthening referral systems

This project focused on the second and last delays in the three-delays model [19]. To gear it up, the availability of transportation was dealt with as the primary strategy. To increase health accessibility to MNCH services and responsiveness, 6 ambulances to Kibaha District, Kibaha Town, Kisarawe District, Rufiji District, Mafia District and Kibiti District were provided.

#### Mentorship programme

Mentorship programme is essential, especially for newly trained associate clinicians responsible for managing the full range of obstetric complications without a more experienced clinician in the facility [13]. The mentorship activities included (1) on-site supervision and mentorship visits, (2) off-site continuing medical education targeting skills improvement to manage identified problems, (3) discussing obstetric cases, sharing challenges and feedback, and (4) a 24-hour on-call system staffed by health providers. Mentorship activities focused on identified areas of substandard care.

#### Strengthening leadership and management

The Regional Health Management Team (RHMT) and the CHMT designed capacity-building and onsite mentorship to equip health managers, including district medical officers (DMOs), with essential knowledge and skills for leading change. These were basic principles and strategies in leading change to improve general health performance and CEmONC services at health facilities. The workshops were conducted at least once a year during the project implementation period. Participants were from 18 facilities and council and regional health management team members. Quality improvement plans were developed by the RHMT in cooperation with the MOH, where mentors or lecturers were dispatched to the health facilities and health management teams and jointly addressed the identified gaps (Table 1).

**Table 1:** Project interventions and scope

Interventions	Scope
1. Decentralising CEmONC Services	
Facility Upgrade (Construction, Renovation, Refurbishment)	7 HC upgraded - Operating theatres and Maternity ward - Laboratory and other renovations - Supplying essential medical equipment - Equipping WASH & IDPC - Power supply (Solar panels, Generators) - Blood bank availability
Training to deliver CEmONC (Medical Doctors and Nurses)	1 RRH, 10 HCs, and 3 DHs - CEmONC (HC & DH) - Theatre Management Training - Anaesthesia Training & Deployment (Nurses) - IDPC Training (Medical Doctors & Nurses)
**2. Strengthening Transportation for Strengthening Referral Systems**	
Provision of Ambulances	6 ambulances provided to 6 councils - Kibaha District, Kibaha Town, Kisarawe District, Rufiji District, Mafia District and Kibiti District
Support Referral System among HFs	1 RRH - Setting up referral systems and referral drills to NH
**3. Support High Quality EmONC at the PHC facilities**	
Equipment and Renovation	1 RRH, 3 DHs, 10 HCs, and 5 DSPs - Construction & Renovation of New Theatre - Construction & Renovation of Maternity Ward - Procurement of Essential Medical Equipment
Training to deliver BEmONC	All but DSP-centred - BEmONC Service Quality (DSP) - KMC, Abnormal Pregnancy Signs Training - DSPs Renovation for routine OBGYN Care
Other Trainings & Activities	3 DHs, 10 HCs, and 5 DSPs - Blood Donation & Sensitisation - Village Health Day - HPV Announcement & Sensitisation - Biomedical equipment repairing - CHWs Training (2 CHWs per a DSP)
**4. Mentorship Programme**	
RHMT, CHMT and MOI level Supervision and Mentorship	10 HCs, and 5 DSPs - Mentors or Lecturers visited and offered In-Service Training (In cooperation with the MOH)
**5. Strengthening Leadership and Management**	
Strengthen Accountability for good-Quality Service Delivery	CHMT level - Recognising the Best Performance Council (Yearly)
Mentoring Plan, Budget and Manage	CHMT and RHMT level

†*Abbreviations*: *CEmONC* Comprehensive Emergency Obstetric and Newborn Care, *CHMT* Council Health Management Team, *CHW* Community Health Worker, *DH* District Hospital, *DSP* Dispensary, *HC* Health Centres, *HF* Health Facility, *IDPC* Infectious Disease Prevention & Control, *MOH* Ministry of Health, *MOI* Medical Officer In Charge, *NH* National Hospital, *OBGYN* Obstetrics & Gynaecology, *PHC* Primary Health Care, *RHMT* Regional Health Management Team, *RH* Regional Referral Hospital, *WASH* Water & Sanitation

### Data collection

#### DHIS2 data collection from intervention and control groups

All data from both intervention and control groups were manually extracted from the DHIS2 covering from 2016 to 2022. Regarding missing data, at first, the Regional Reproductive and Child Health Coordinator and the Project Coordinator followed up together by collating internal secondary documents such as existing project reports and monitoring reports. Secondly, nevertheless, missing data unsolved were treated with the median value. From the DHIS2, ‘ANC 4 + Rate (%)’, ‘Per cent of Mothers tested Anaemia during ANC’, ‘Caesarean Section Delivery Rate (%)’, ‘Per cent of Mothers and Newborns who received PNC services within 48 hours’, ‘Delivery Complication Rate (%)’, and ‘SBA Delivery Rate (%)’ were used as dependent variables in consideration of the intervention, and data quality and validity.

#### Facility data from the intervention group

Facility data were collected by collating with existing project reports, such as monitoring reports and infrastructure development reports. A self-administered health facility physical status assessment tool was used for facility infrastructure data collection during monitoring field trips [17].

### Statistical analysis

The statistical analysis was conducted at the PHC level only due to data homogeneity. To summarise and analyse outcomes derived from the DHIS2 and health facility assessments, a range of statistical tests were utilised, including descriptive statistics (e.g., frequency and percentage), and inferential statistics (e.g., paired sample t-test, one-way ANOVA, chi-square (χ2) test, Fisher’s exact test, and Pearson correlation test). In the first instance, we performed descriptive analyses to explore the distribution of outcome metrics per facility in both intervention and control groups. Next, we used the paired sample t-test to compare the impact on MNCH variables on before and after intervention results in both intervention and control health facilities. Prior to analysing the paired sample t-test, the F-test was conducted to compare variances. And then, we did the one-way ANOVA to assess the impact of the intervention model by comparing dependent variables as outcomes between intervention and control groups. The Tukey-Kramer test was used for post hoc analysis to assess differences among groups. The χ2 test was applied to assess the statistical significance of differences in upgraded HCs for Caesarean Section Delivery (%). Additionally, χ2 analysis was employed to examine the statistical significance between variables and the physical status of each PHC facility that underwent upgrading due to the project. When the expected frequency is below 5, the χ2 test may not be reliable; therefore, verification was conducted using Fisher’s exact test. Health facility status variables include (1) Type of health facility, (2) Distance from the nearest facilities, (3) Years of operation, (4) Catchment population, (5) Functionality of CEmONC, (6) Water availability, (7) Electric power availability, (8) Phone availability, (9) Internet network availability, (10) Incinerator availability, 11) Ash pit availability, 12) Placenta pit availability, 13) Staff house availability, 14) Blood bank availability, 15) Waste bin availability for IDPC, 16) Physical status of the facility categorised into (1) to (6), and 17) Ambulance availability for referral system. Amenities (variables No. 6 to 15 and 17) variables were measured by binary response format. Further, the Pearson correlation test was used for significance between funding size and the change of MNCH variables. Statistical significance at 0.05 was calculated by using Microsoft Excel^®^ 2013. Coordinates were manually collected by mobile phone during monitoring field trips, and it was used for mapping. The site map was created by QGIS version 3.30.

## Results

### Demographic information and health facility improvement

There were 19 public health facilities (5 DSPs, 10 HCs, 3 DHs and 1 RRH) supported and refurbished (*However*,* RRH was excluded in the analysis*). Of these health facilities, 15 (83%) were in rural settings, whilst 3 (17%) were in urban areas. All 5 DSPs were in hard-to-reach places in a small island (Mafia Island) where only BEmONC services were provided. Totally 7 HCs (70%) out of 10 were upgraded where Caesarean Section Delivery and Blood Bank services are accessible. There were 2 (66%) out of 3 DHs in urban settings, and the remaining one was in Mafia Island. Major health facility upgrades and rehabilitation components were MNCH-centred infrastructure installation, including 6 maternity theatres, 9 maternity labour and delivery wards, 1 laboratory, 2 3-in-1 incinerators, 5 reproductive and child health clinics, 1 facility-associated infection laundry building and the provision of medical equipment.

### CEmONC preparedness and availability through Decentralisation

**Fig. 3:**
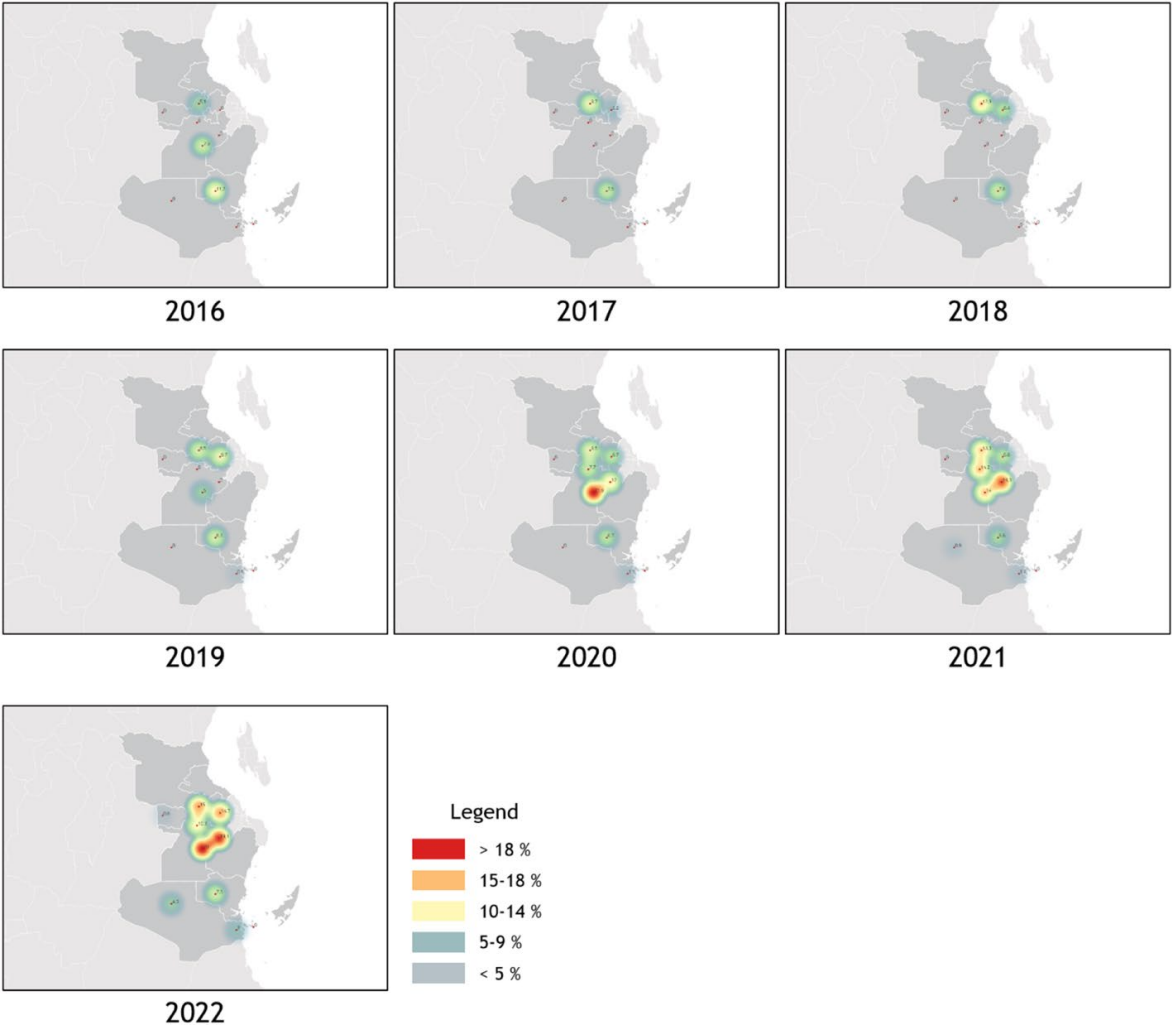
A series of visualised maps of Caesarean Section Delivery Coverage in Pwani intervention group from 2016 to 2022

Beginning in 2017, the deployment of maternity theatres, anaesthesia machines, and a skilled workforce - including medical doctors and nurses - was initiated. In 2016, only 3 HCs provided CEmONC services, and as of 2022, Caesarean Section Delivery became available in all 10 HCs within the intervention group (Fig. 3). During the same period, the control groups also experienced an increase in CEmONC availability, in Pwani control rising from 0 HCs in 2016 to 9 HCs in 2022, in Dodoma control increasing 2 HCs in 2016 to 5 HCs in 2022. However, the observed increase in the intervention group did not reach statistical significance compared to the control groups (χ2 = 2.82, *p* = 0.2448, df = 2).

### Impact assessment by the paired sample t-test on MNCH variables on before and after intervention

The mean ANC 4 + Rate of 18 facilities in Pwani intervention group in 2016 was 16.96%, then it increased to 32.43% in 2022 (*n* = 18, t= -6.51, ****p < 0.0001 (95% CI: -20.49, -10.46)). The mean per cent of Mothers tested Anaemia during ANC as an indicator for ANC quality services was increased from 58.79 to 93.73% (*n* = 18, t= -4.54, ****p < 0.0001 (95% CI: -51.17, -18.71)). While the mean Caesarean Section Delivery Rate was 5.54% at the beginning of the intervention, it was achieved an average of 14.06% in 2022 (*n* = 13, t= -2.22, *p = 0.0382 (95% CI: -16.89, -0.16)). We also found that other MNCH variables were increased during the same period. However, there was no statistical significance in such changes (PNC within 48 h, Mothers: *n* = 18, t= -1.74, *p* = 0.0920 (95% CI: -40.80, 3.95)) / Newborns: *n* = 18, t= -1.76, *p* = 0.0874 (95% CI: -40.20, 3.65)), Delivery Complication Rate (*n* = 13, t= -0.79, *p* = 0.4364 (95% CI: -4.43, 2.07)) and SBA Delivery Rate (*n* = 18, t= -1.61, *p* = 0.1175 (95% CI: -8.24, 1.11)) (Table 2). Meanwhile, in the control groups, MNCH variables were shown to have better outcomes than Pwani intervention group in the same period, except for the Delivery Complication Rate variable. The Delivery Complication Rate variable has no significant change between baseline and endline in all intervention and control groups.

**Table 2:** A paired sample t-test result on MNCH variables (before (2016) and after (2022))

			Pwani Intervention					(*P*) Control					(*D*) Control				
Variable	Pre/ Post	*n*	Mean	SD	t	*p* < 0.05	95% CI	Mean	SD	t	*p* < 0.05	95% CI	Mean	SD	t	*p* < 0.05	95% CI
ANC 4 + Rate (%)	Pre	18	16.96	3.71	-6.51	*p* < 0.0001	-20.49, -10.46	20.97	5.33	-4.19	0.0002	-15.39, -5.08	20.09	9.56	-2.57	0.0148	-16.02, -1.86
	Post	18	32.43	9.07				31.21	8.55				29.04	10.72			
% of Mothers received Anaemia test during ANC	Pre	18	58.79	30.89	-4.54	*p* < 0.0001	-51.17, -18.71	59.80	36.24	-1.33	0.1922	-39.47, 8.94	83.58	23.45	-2.33	0.0302	-25.84, -1.78
	Post	18	93.73	7.20				75.06	30.38				97.39	6.76			
C-Sec Delivery Rate (%)	Pre	13	5.54	6.90	-2.22	0.0382	-16.89, -0.16	2.59	6.58	-2.88	0.0099	-20.80, -2.89	7.88	12.11	-0.72	0.4766	-14.02, 6.75
	Post	13	14.06	11.37				14.44	12.62				11.52	12.54			
% of Mothers received PNC within 48 h	Pre	18	69.92	34.74	-1.74	0.0920	-40.80, 3.95	66.14	31.82	-2.13	0.0407	-42.94, -0.98	50.46	33.07	-5.97	*p* < 0.0001	-64.56, -31.79
	Post	18	88.34	26.55				88.11	28.27				98.63	3.38			
% of Newborns received PNC within 48 h	Pre	18	69.62	33.65	-1.76	0.0874	-40.20, 3.60	66.42	36.52	-2.34	0.0251	-53.03, -3.78	52.04	35.00	-5.44	*p* < 0.0001	-64.89, -29.59
	Post	18	87.92	26.44				94.83	34.09				99.28	7.53			
Delivery Complication Rate (%)	Pre	13	4.87	3.06	-0.79	0.4364	-4.43, 2.07	4.93	6.41	-0.43	0.6730	-7.80, 5.12	4.31	4.32	-0.48	0.6328	-3.88, 2.40
	Post	13	6.06	4.16				6.92	9.13				4.91	3.34			
SBA Delivery Rate (%)	Pre	18	92.18	7.24	-1.61	0.1175	-8.24, 1.11	91.48	12.12	1.02	0.3152	-4.65, 14.01	81.38	17.59	-3.57	0.0020	-24.77, -6.80
	Post	18	95.74	5.58				86.79	14.55				97.17	4.77			

†*Abbreviations*: *(P) Control* Pwani Control, *(D) Control* Dodoma Control, *ANC* Antenatal Care, *CI* Confidence Interval, *C-Sec* Caesarean Section, *PNC* Postnatal Care, *SBA* Skilled Birth Attendance, *SD* Standard Deviation

### Results of one-way ANOVA among intervention and control groups

We evaluated the significance of the dependent variables using one-way ANOVA with control groups as a reference. The majority of changes in MNCH variables did not achieve statistical significance, with the exception of the ‘Percentage of Mothers Tested for Anaemia during ANC’ (****p < 0.0001), ‘Percentage of Newborns Receiving PNC Services within 48 Hours’ (**p = 0.0095), and the ‘SBA Delivery Rate’ (***p = 0.0043). To further assess these mean differences between the pre-intervention period (2016) and the post-intervention period (2022), the Tukey-Kramer test was employed as a post hoc analysis. The results of the Tukey-Kramer test confirmed that the differences between 2016 and 2022 were statistically significant at the *p*-value < 0.05 level. At the same time, this method was also used for homogeneity among groups, and it showed that there were no statistical differences in homogeneity in the pre-intervention period by *p*-value > 0.05 (Table 3).

**Table 3:** The one-way ANOVA table for the impact assessment on MNCH variables

Variable	Year of 2016							Year of 2022							
ANC 4 + Rate (%)		**count**	**sum**	**mean**	**variance**				**count**	**sum**	**mean**	**variance**			
	Intervention	18	305.20	16.96	14.54			Intervention	18	583.8	32.43	87.08			
	(P) Control	18	377.50	20.97	30.13			(P) Control	18	561.7	31.21	77.35			
	(D) Control	18	361.70	20.09	96.76			(D) Control	18	522.7	29.04	121.73			
		***ss***	***df***	***ms***	***F***	*p* < 0.05	***F crit***		***ss***	***df***	***ms***	***F***	*p* < 0.05	***F crit***	
	B/W groups	160.54	2	80.27	1.70	0.1924	3.18	B/W groups	106.34	2	53.17	0.56	0.5761	3.18	
	W/In groups	2404.3	51	47.143				W/In groups	4864.61	51	95.38				
	*Tukey-Kramer test*		*Q critical value: 5.54*			*p* > 0.05				*Q critical value: 7.87*			*p* > 0.05		
% of Mothers tested Anaemia during ANC		**count**	**sum**	**mean**	**variance**				**count**	**sum**	**mean**	**variance**			
	Intervention	18	1058.20	58.79	1010.17			Intervention	18	1687.1	93.73	54.86			
	(P) Control	18	1076.35	59.80	1390.97			(P) Control	18	1076.35	59.80	1390.97			
	(D) Control	18	1504.50	83.58	582.45			(D) Control	18	1753.1	97.39	48.32			
		***ss***	***df***	***ms***	***F***	*p* < 0.05	***F crit***		***ss***	***df***	***ms***	***F***	*p* < 0.05	***F crit***	
	B/W groups	7089.36	2	3544.68	3.56	0.0356	3.18	B/W groups	15469.67	2	7734.83	15.53	0.0000	3.18	
	W/In groups	50721.10	51	994.53				W/In groups	25400.50	51	498.05				
	*Tukey-Kramer test*		*Q critical value: 25.42*			*p* > 0.05				*Q critical value: 17.99*			*p* < 0.05		
C-Sec Delivery Rate (%)		**count**	**sum**	**mean**	**variance**				**count**	**sum**	**mean**	**variance**			
	Intervention	13	72.00	5.54	51.52			Intervention	13	182.80	14.06	140.16			
	(P) Control	13	33.70	2.59	46.97			(P) Control	13	187.69	14.44	172.55			
	(D) Control	13	102.50	7.88	158.82			(D) Control	13	149.80	11.52	170.36			
		***ss***	***df***	***ms***	***F***	*p* < 0.05	***F crit***		***ss***	***df***	***ms***	***F***	*p* < 0.05	***F crit***	
	B/W groups	182.84	2	91.42	1.07	0.3551	3.26	B/W groups	65.35	2	32.67	0.20	0.8173	3.26	
	W/In groups	3087.78	36	85.77				W/In groups	5796.92	36	161.03				
	*Tukey-Kramer test*		*Q critical value: 9.59*			*p* > 0.05				*Q critical value: 13.14*			*p* > 0.05		
% of Mothers received PNC within 48 H		**count**	**sum**	**mean**	**variance**				**count**	**sum**	**mean**	**variance**			
	Intervention	18	1258.50	69.92	1277.95			Intervention	18	1590.10	88.34	746.34			
	(P) Control	18	1190.60	66.14	1071.94			(P) Control	18	1585.90	88.11	846.42			
	(D) Control	18	908.20	50.46	1157.94			(D) Control	18	1775.30	98.63	12.08			
		***ss***	***df***	***ms***	***F***	*p* < 0.05	***F crit***		***ss***	***df***	***ms***	***F***	*p* < 0.05	***F crit***	
	B/W groups	3834.63	2	1917.32	1.64	0.2041	3.18	B/W groups	1299.80	2	649.90	1.21	0.3052	3.18	
	W/In groups	59632.97	51	1169.27				W/In groups	27282.29	51	534.95				
	*Tukey-Kramer test*		*Q critical value: 27.57*			*p* > 0.05		*Tukey-Kramer test*		*Q critical value: 18.65*			*p* > 0.05		
% of Newborns received PNC within 48 H		**count**	**sum**	**mean**	**variance**				**count**	**sum**	**mean**	**variance**			
	Intervention	18	1253.10	69.62	1198.96			Intervention	18	1582.50	87.92	740.5			
	(P) Control	18	1195.60	66.42	1412.33			(P) Control	18	1195.60	66.42	1412.33			
	(D) Control	18	936.70	52.04	1297.09			(D) Control	18	936.70	52.04	1297.09			
		***ss***	***df***	***ms***	***F***	*p* < 0.05	***F crit***		***ss***	***df***	***ms***	***F***	*p* < 0.05	***F crit***	
	B/W groups	3156.38	2	1578.19	1.21	0.3062	3.18	B/W groups	11736.60	2	5868.32	5.10	0.0095	3.18	
	W/In groups	66442.50	51	1302.79				W/In groups	58646.20	51	1149.93				
	*Tukey-Kramer test*		*Q critical value: 29.08*			*p* > 0.05				*Q critical value: 27.33*			*p* < 0.05		
Delivery Complication Rate (%)		**count**	**sum**	**mean**	**variance**				**count**	**sum**	**mean**	**variance**			
	Intervention	13	63.36	4.87	10.17			Intervention	13	78.72	6.06	18.72			
	(P) Control	13	66.84	5.14	41.71			(P) Control	13	84.26	6.48	85.74			
	(D) Control	13	54.23	4.17	18.92			(D) Control	13	63.81	4.91	11.13			
		***ss***	***df***	***ms***	***F***	*p* < 0.05	***F crit***		***ss***	***df***	***ms***	***F***	*p* < 0.05	***F crit***	
	B/W groups	6.53	2	3.26	0.14	0.8714	3.26	B/W groups	17.21	2	8.61	0.22	0.8009	3.26	
	W/In groups	849.66	36	22.60				W/In groups	1387.07	36	38.53				
	*Tukey-Kramer test*		*Q critical value: 5.34*			*p* > 0.05				*Q critical value: 6.43*			*p* > 0.05		
SBA Delivery Rate (%)		**count**	**sum**	**mean**	**variance**				**count**	**sum**	**mean**	**variance**			
	Intervention	18	1659.20	92.18	55.50			Intervention	18	1723.40	95.74	32.98			
	(P) Control	18	1646.60	91.48	155.46			(P) Control	18	1562.30	86.79	224.06			
	(D) Control	18	1464.90	81.38	81.38			(D) Control	18	1749.00	97.17	24.12			
		***ss***	***df***	***ms***	***F***	*p* < 0.05	***F crit***		***ss***	***df***	***Ms***	***F***	*p* < 0.05	***F crit***	
	B/W groups	1313.45	2	656.72	3.66	0.0327	3.18	B/W groups	1138.25	2	569.12	6.07	0.0043	3.18	
	W/In groups	9154.47	51	179.50				W/In groups	4779.77	51	93.72				
	*Tukey-Kramer test*		*Q critical value: 10.08*			*p* > 0.05				*Q critical value: 7.80*			*p* < 0.05		

†*Abbreviations*: *(P) Control* Pwani Control, *(D) Control* Dodoma Control, *ANC* Antenatal Care, *B/W groups* Between groups, *C-Sec* Caesarean Section, *df *Degree of freedom, *F crit * F critical value, *ms* Mean Squares, *PNC* Postnatal Care, *SBA* Skilled Birth Attendance, *ss* Sum of Squares, *W/In groups* Within groups

### DHFF funding size and changes in MNCH variables

We conducted a Pearson correlation analysis to assess the relationship between the DHFF funding size and changes in MNCH variables. The analysis revealed no statistically significant correlations. The R² values for each variable were as follows: ANC 4 + Rate (0.0021), Percentage of Mothers Tested for Anaemia during ANC (0.0082), Caesarean Section Delivery Rate (0.1280), Percentage of Mothers and Newborns Receiving PNC within 48 h (0.0016 and 0.0002, respectively), Delivery Complication Rate (0.1545), and SBA Delivery Rate (0.1619). The corresponding Pearson correlation coefficients for these variables were calculated as -0.0458, 0.0905, 0.3577, -0.0406, -0.0156, 0.4023, and 0.3930, respectively.

### Primary health facility functionality and statistical correlations

Health facility level was significantly associated by using bivariate analysis (χ2) (*p = 0.0347) (Table 4). We also unveiled that the distance to health facilities is positively associated with the location of facilities (****p < 0.0001), as people who dwell in urban areas have access to their facilities within 5 km as opposed to those who are in the rural counterparts. There are 13 (72%) out of 18 facilities provided CEmONC services, including blood bank and transfusion (*the remaining 5 facilities are DSPs which deliver BEmONC only in accordance with the health cadres*). Even though the importance of a staff house has been emphasised for 24-hour on-call purposes, most PHC facilities are still in need. Ambulances for patient transfer are also insufficient at half of the facilities. 61% (*n* = 11), 89% (*n* = 11), and 78% (*n* = 14) of facilities have available water supply (either piped or borehole), electric power supply, and telecommunication (either mobile or landline), respectively, while internet connectivity was only 28% (*n* = 5) and significantly associated (*p = 0.0186). During the COVID-19 pandemic, PHC-based IDPC functionalities were strengthened. As a result, 94% (*n* = 17) of facilities were equipped with 3-in-1 incinerators, 67% (*n* = 12) had ash pits, and all facilities (*n* = 18) had placenta pits and waste bins. All assessed health facilities have been operational for more than 10 years (*n* = 18). Despite this, 39% (*n* = 7) of the facilities were in good (A) or moderate (B) condition, while 55% (*n* = 10) required significant renovation efforts.

**Table 4:** Descriptive results of Primary Health facilities by geographical settings (Urban and Rural) in Pwani region (*n* = 18) by 2022

Variable	Description	Rural *n* (%)	Urban *n* (%)	Total (%)	χ2	df	*p* < 0.05
HF level*	DSP	5 (33)	0 (0)	5 (28)	6.72	2	*0.0347*
	HC	9 (60)	1 (33)	10 (56)			
	DH	1 (7)	2 (67)	3 (16)			
Distance from the nearest HF (km)	< 5	0 (0)	3 (100)	3 (17)	18.00	2	*< 0.0001*
	5–10	12 (80)	0 (0)	12 (66)			
	11–20	3 (20)	0 (0)	3 (17)			
Catchment Population	< 5,000	7 (47)	0	7 (39)	5.04	4	0.2832
	5,001–10,000	2 (13)	0	2 (11)			
	10,001–15,000	1 (7)	1 (33)	2 (11)			
	15,001–20,000	4 (26)	1 (33)	5 (28)			
	> 20,000	1 (7)	1 (33)	2 (11)			
Year of Operation†	< 5	0 (0)	0 (0)	0 (0)	N/A	N/A	N/A
	5–10	0 (0)	0 (0)	0 (0)			
	10+	15 (100)	3 (100)	18 (100)			
Training Status‡	< 50%	14 (93)	3(100)	17 (94)	0.00	1	1.0000
	> 50%	1 (7)	0 (0)	1 (6)			
CEmONC Service Provision‡	Y	10 (67)	3 (100)	13 (72)	0.22	1	0.6379
	N	5 (33)	0 (0)	5 (28)			
CEmONC Preparedness							
Staff House‡	Y	4 (27)	0 (0)	4 (22)	0.06	1	0.7998
	N	11 (73)	3 (100)	14 (78)			
Blood Bank‡	Y	10 (67)	3 (100)	13 (72)	0.22	1	0.6379
	N	5 (33)	0 (0)	5 (28)			
Ambulance‡	Y	6 (40)	3 (100)	9 (50)	1.60	1	0.2059
	N	9 (60)	0 (0)	9 (50)			
Functional Amenities							
Water‡	Y	8 (53)	3 (100)	11 (61)	0.75	1	0.3871
	N	7 (47)	0 (0)	7 (39)			
Electric Power‡	Y	13 (87)	3 (100)	16 (89)	0.00	1	1.0000
	N	2 (13)	0 (0)	2 (11)			
Phone‡	Y	11 (73)	3 (100)	14 (78)	0.06	1	0.7998
	N	4 (27)	0 (0)	4 (22)			
Internet Network‡	Y	2 (13)	3 (100)	5 (28)	5.54	1	*0.0186*
	N	13 (87)	0 (0)	13 (72)			
Medical Waste Management							
Incinerator‡	Y	14 (93)	3 (100)	17 (94)	0.00	1	1.0000
	N	1 (7)	0 (0)	1 (6)			
Ash Pit‡	Y	9 (60)	3 (100)	12 (67)	0.45	1	0.5023
	N	6 (40)	0 (0)	6 (33)			
Placenta Pit†‡	Y	15 (100)	3 (100)	18 (100)	N/A	N/A	N/A
	N	0 (0)	0 (0)	0 (0)			
Waste Bin for IDPC†‡	Y	15 (100)	3 (100)	18 (100)	N/A	N/A	N/A
	N	0 (0)	0 (0)	0 (0)			
Physical Status†§	A	2 (13)	1 (33)	3 (17)	1.68	3	0.6414
	B	4 (27)	0	4 (22)			
	C	8 (53)	2 (67)	10 (55)			
	D	0	0	0 (0)			
	E	1 (7)	0	1 (6)			
	F	0	0	0 (0)			

*Abbreviations: *CEmONC* Comprehensive Emergency Obstetric and Newborn Care, *df* Degree of freedom, *DH* District Hospital, *DSP* Dispensary, *HC* Health Centre, *HF* Health Facility, *IDPC* Infectious Disease Prevention & Control

† Variables with a frequency of 0 in the ‘*N*’ category in observed values are excluded from the analysis

‡ Fisher’s exact test was used for the verification since more than 25% of the expected frequencies are below 5

§ Physical Status Definition: (A) Good = A building with nothing to be fixed, (B) Minor Renovation= 1) Visible narrow crack on concrete surface (< 0.2 mm), 2) Damage to the door locks, 3) Damage to the window glass, (C) Major Renovation= 1) Visible wide crack (0.2 mm ~ 1.0 mm), 2) Foundation damage, 3) Significant damage to concrete (exposed reinforcing bar), 4) Spalling of concrete surface, (D) Demolition and Reconstruction= 1) Foundation damage, 2) Buckling of reinforcing bars, 3) Cracks in core concrete, 4) Visible vertical and/or lateral deformation in columns and/or walls, 5) Visible settlement and/or leaning of the building, (E) Under Construction= 1) From the laying of the foundation to the final stage, (F) Under Renovation= 1) Any rehab activity

## Discussion

This six-year project focused on improving the quality of MNCH services, primarily by strengthening and decentralising CEmONC services through the DHFF model at the PHC level. The project aimed to refurbish and renovate health infrastructure, procure equipment, and enhance the capacity of health care providers. A significant portion of the funds (approximately 70%) was allocated to upgrading health facilities and providing medical equipment, in alignment with GoT’s health sector strategic plan, as corroborated by previous studies [17, 20]. The DHFF initiative in Pwani region enhanced access and equity in public PHC infrastructure development, contributing to the goal of achieving UHC [15]. The project had a positive impact on MNCH indicators, particularly in expanding CEmONC coverage, aligning with the GoT’s objective of decentralising CEmONC services and ensuring that at least 80% of HCs provide CEmONC services across the country [12].

In our impact assessment, specific MNCH indicators, such as the ‘Percentage of Mothers tested for Anaemia during ANC,’ showed significant improvement compared to both Pwani and Dodoma controls, which we used as a measure of ANC quality services. Additionally, the ‘Percentage of Newborns receiving PNC within 48 hours’ was statistically significant, despite the paired sample t-test not reaching statistical significance (*p* = 0.0874), the one-way ANOVA result indicated a significant change (**p = 0.0095) when compared with the Dodoma control. From the analysis of PHC functionality, we found that the level of the health facilities (DSP, HC, DH was significant (*p = 0.0347). We also uncovered that the distance to health facilities was significantly associated with the location (rural or urban) of the facilities (****p < 0.0001), with urban residents having better access within 5 km compared to their rural counterparts. This finding is consistent with previous studies [17].

Beyond the statistical significance of the MNCH variables, one unquantifiable impact of the project was the MNCH continuum services during the COVID-19 pandemic. The DHIS2 data indicated that most PHC facilities maintained similar levels of MNCH service provision compared to the pre-pandemic period (*data not shown*). During this time, the functionality of PHC-based IDPC measures, such as incinerators, ash pits, placenta pits, and waste bins, was also enhanced. This was significant in leveraging health governance expertise and strengthening accountability among partners, contributing to the achievement of SDG 3 and 17 [21–23].

Decentralising CEmONC services by upgrading and scaling up health facilities has been a key strategy of the health authorities in Tanzania [24]. Over the past decade, numerous studies have highlighted the positive impact of decentralising CEmONC service delivery on MNCH outcomes [13, 15, 25–27]. These studies have documented various benefits, including increased institutional deliveries, ANC4 + coverage, Caesarean deliveries, and reduced adverse pregnancy outcomes, maternal and neonatal deaths [15, 24, 26].

Despite having these successes in the previous studies, our research was not initially designed as a controlled intervention study. For evaluation purposes, we confirmed the absence of significant differences in MNCH variables between intervention and control groups in 2016 using the Tukey-Kramer method. The subsequent impact assessment revealed some limitations. Although all MNCH indicators showed increases, most did not reach statistical significance when compared with control groups (ANC 4 + Rate, Caesarean Section Delivery Rate, Percentage of Mothers receiving PNC services within 48 h, Delivery Complication Rate, SBA Delivery Rate). The expansion of CEmONC coverage was a notable outcome, but not all HCs (only 9 out of 10) were able to provide CEmONC services during the project. We hypothesise that factors such as misconceptions, poor accessibility, and a shortage of health care personnel were likely to be influenced outcomes [28–30]. The impact of expanding CEmONC had also no statistical significance in comparison with control groups. From the Pwani control, it increased from 0 HCs in 2016 to 9 HCs in 2022. The number of Caesarean Section Delivery capable HCs was 2 in 2016, but it soared to 5 in the Dodoma control.

Additionally, while the major interventions focused on infrastructure, many facilities still lack essential resources, such as staff housing. Only half of the facilities operate ambulances, with a more pronounced shortage in rural areas (60% of rural facilities lack ambulances). Among the six ambulances procured, three were allocated to urban settings and three to rural areas. Furthermore, 55% of the facilities still require significant renovations, casting doubt on the overall positive impact of the project.

These findings appear to contradict those of previous studies [13, 15, 26–28]. We cautiously suggest that this discrepancy may be due to the project’s implementation modality. Unlike earlier studies in Kigoma, Morogoro, and even in Pwani, which were often directly managed by external organisations, our project was implemented in collaboration with the GoT through the DHFF model. While external management facilitates data collection and prompt decision-making, it can also undermine local health systems and weaken management authority. Therefore, the DHFF approach is fundamentally sound, but it requires strong management skills and governance structures. It is possible that both the implementers and donors initially underestimated the importance of these factors. However, previous studies on the DHFF have demonstrated its effectiveness in health system strengthening when the management and governance structures are well-defined [9, 31–33].

Furthermore, the estimated costs of upgrading an HC to provide CEmONC services in Tanzania are substantial: $256,650 USD for infrastructure and equipment, $4,463 USD per personnel for clinical skills training such as anaesthesia (over three months), and $43,500 USD per year for medicines and supplies [25, 33]. Given the scope of the interventions and the funding size, it appears that these considerations were not fully appraised at the project’s outset. Consequently, we propose two major recommendations for future projects to achieve better outcomes, drawing on the lessons learned from this assessment.

### The economies of scale

Economies of scale can be defined as “*the relationship between the scale of use of a properly chosen combination of all productive services and the rate of output of the enterprise*” [34]. In simpler terms, it describes the optimal scale of activity. For this project, the total funding amounted to approximately 4 million USD over six years, distributed across 19 health facilities. After accounting for overhead and necessary administrative expenditures, the net funding available for direct project activities was roughly 2.2 million USD. This translates to an arithmetic average funding size of 112,416.4 USD per facility, varying based on geographic location, scope, and functionality. The DHFF model may face challenges when resources are limited, particularly if issues such as understaffing, inadequate training, or delays in fund disbursement are not efficiently managed [10, 35]. In this context, the number of target facilities may have been too many to allow for a robust assessment of MNCH effectiveness through the DHFF system. This scale mismatch could have contributed to the poor statistical significance observed in many MNCH variables.

### DHFF data quality management

While the Pwani RHMT generally maintains well-managed data control systems - periodically collecting, collating, updating, and storing fully coded health data - the data management process at the beginning of the project faced significant challenges. Crucial issues, particularly related to data management among stakeholders, including the funder, were not adequately addressed. This oversight led to substantial missing data in key outcome variables, severely limiting the use of panel data and hindering comprehensive analysis of various outcome variables. As this assessment was conducted retrospectively, it became evident that critical aspects of data management - including collection, validation, and monitoring - were likely underestimated during the planning and implementation stages. Given that the project spanned several years, the project steering committee should have established a dedicated and rigorous data management scheme from the outset. This would have mitigated the issues related to missing data and allowed for a more thorough analysis of the project’s impact.

### Limitations

There are two limitations of this research worth highlighting. First, while the contribution of the DHFF to MNCH indicators was assessed throughout the project’s implementation period, there may be a disconnect between the observed data and the reality on the ground. Several studies have demonstrated that the DHFF model positively influences health governance, workforce capacity, and the availability of health commodities from a health systems perspective, with strong community stakeholder engagement [10, 36–38]. However, since the scope of the project was limited to the health provider level, its impact on community stakeholders remains unexplored. To verify the practical impact of the project on communities, a comprehensive community-based survey, including both quantitative and qualitative approaches, should be conducted. The roles of health providers at health facilities and community leaders are critical in managing and executing DHFF activities, such as planning and budgeting, which are directly linked to the quality of care provided [31–33]. Second, despite the project’s limited impact on MNCH variables, it is worth noting that MNCH indicators in Tanzania have generally improved over the past decade. This assessment suggests that the expansion of CEmONC coverage and decentralisation strategies have been effective. However, due to financial constraints, this research was confined to 19 health facilities within a single region (Pwani) out of the 26 regions in Tanzania Mainland. Consequently, the findings cannot be generalised beyond Pwani region. To provide a more holistic picture of the DHFF system’s functionality, future studies should be conducted with broader geographical coverage, encompassing more regions, and extending the period of observation. This would allow for a more comprehensive understanding of the DHFF’s impact on MNCH and potentially other health outcomes in Tanzania.

## Conclusions

Our evaluation of the Health System Strengthening Project, which focused on MNCH services through the DHFF model in Pwani region, revealed that the project was limited functional and partially contributed to improving MNCH services. Notably, the indicators for ‘Percentage of Mothers Tested for Anaemia during ANC,’ ‘Percentage of Newborns Receiving PNC within 48 Hours,’ and ‘SBA Delivery Rate’ showed significant improvements. Furthermore, paired sample t-test results indicated significant changes in the ANC 4 + Rate and Caesarean Section Delivery Rate. However, these results were not supported by the one-way ANOVA and Tukey-Kramer tests, and the increase in CEmONC coverage, while positive, lacked statistical significance.

Our analysis did not find a significant correlation between the size of DHFF funds and changes in MNCH variables. However, the functionality of the PHC facilities was positively influenced by factors such as facility level (DSP, HC, DH; *p = 0.0347), proximity to health facilities (****p < 0.0001), and internet connectivity (*p = 0.0186). Despite these advancements, many PHC facilities continue to require substantial renovations and improvements in WASH, internet, and power supply.

To enhance the impact on MNCH variables, we recommend considering the economies of scale in project design, as well as critically examining the DHFF management and governance structures. Apart from statistical significance, this project successfully leveraged the expertise of both health and local government authorities and strengthened accountability among partners, contributing to the achievement of SDG 3 and 17. In particular, district councils supervised construction activities, engaging local labour and ensuring community ownership, while MNCH services continued uninterrupted during the COVID-19 pandemic, with improved PHC-based IDPC functionalities.

Moving forward, we suggest two critical areas for improvement. Firstly, the economies of scale viewpoint regarding the selection of the facilities should be critically considered. Second, the data management scheme should align with the project life cycle, including baseline, midline, and endline. Furthermore, a supply of enough information management devices such as computers and a strengthening availability of stable internet and electricity to the PHC facilities is highly needed.

In conclusion, we found that the project had limited impact on improving MNCH variables in terms of statistical significance, even though all indicators were ameliorated. Nonetheless, the DHFF system remains a promising approach for health system strengthening in Tanzania. To achieve better outcomes, it is essential to critically evaluate and strengthen the DHFF management and governance structures before implementation.

## Data Availability

The data and material used for this paper are available from the corresponding author (Kyoung Kyun Oh / kyoungkyun@kofih.org, k.oh.1@research.gla.ac.uk) on reasonable request in writing.

## Abbreviations

BEmONC: Basic Emergency Obstetric and Newborn Care
CEmONC: Comprehensive Emergency Obstetric and Newborn Care
DH: District Hospital
DHFF: Direct Health Facility Financing
DHIS2: District Health Information Software 2
DSP: Dispensary
GoT: Government of Tanzania
HC: Health Centre
IDPC: Infectious Disease Prevention and Control
IMR: Infant Mortality Rate
KOFIH: Korea Foundation for International Healthcare
MMR: Maternal Mortality Ratio
MNCH: Maternal, Neonatal, and Child Health
MOH: Ministry of Health
NH: National Hospital
NM: Neonatal Mortality
PHC: Primary Health Care
PO-RALG: President’s Office, Regional Administration and Local Government
RHMT: Regional Health Management Team
RRH: Referral Hospital
SDG: Sustainable Development Goal
SH: Specialised Hospital
ToC: Theory of Change
U5M: Under-5 Mortality Rate
UHC: Universal Health Coverage
ZH: Zonal Hospital

## References

[1] URT, Demographics. Health & Infant Mortality - UNICEF DATA. URT. https://data.unicef.org/country/tza/. Accessed 19 Sep 2023.

[2] URT. Tanzania’s 2023 Voluntary National Review (VNR) Report on the Implementation of the 2023 Agenda for Sustainable Development. URT. https://www.mof.go.tz/uploads/documents/en-1689095112-Tanzania%20VNR%20FINAL%202023%20including%20statistical%20annex%20(1)_compressed.pdf. Accessed 19 Oct 2023.

[3] MOH. Tanzania Demographic and Health Survey and Malaria Indicator Survey 2022 Key Indicators Report. NBS, OCGS, ICF. https://dhsprogram.com/pubs/pdf/PR144/PPR144.pdf. Accessed 17 Oct 2024.

[4] TNBS. The 2022 Population and Housing Census: Administrative Units Population Distribution Report; Tanzania Mainland. URT. https://www.nbs.go.tz/index.php/en/census-surveys/population-and-housing-census/852-2022-population-and-housing-census-administrative-units-population-distribution-and-age-sex-reports. Accessed 29 Oct 2023.

[5] Kassebaum NJ, Bertozzi-Villa A, Coggeshall MS, Shackelford KA, Steiner C, Heuton KR, et al. Global, regional, and national levels and causes of maternal mortality during 1990–2013: a systematic analysis for the global burden of Disease Study 2013. Lancet. 2014;384:980–1004. 24797575 10.1016/S0140-6736(14)60696-6 PMC4255481

[6] Nyamtema A, Mwakatundu N, Dominico S, Kasanga M, Jamadini F, Maokola K, et al. Introducing eHealth strategies to enhance maternal and perinatal health care in rural Tanzania. Matern Health Neonatol Perinatol. 2017;3:3. 28116114 10.1186/s40748-017-0042-4 PMC5244514

[7] Hanson C, Gabrysch S, Mbaruku G, Cox J, Mkumbo E, Manzi F, et al. Access to maternal health services: geographical inequalities, United Republic of Tanzania. Bull World Health Organ. 2017;95:810–20. 29200522 10.2471/BLT.17.194126 PMC5710083

[8] WHO. Monitoring the Building Blocks of Health Systems. WHO. 2010. https://cdn.who.int/media/docs/default-source/service-availability-and-readinessassessment%28sara%29/related-links-%28sara%29/who_mbhss_2010_cover_toc_web.pdf. Accessed 22 Jul 2023.

[9] Ruhago GM, John MB, Ngalesoni FN, Msasi D, Kapologwe N, Kengia JT, et al. Understanding the implication of direct health facility financing on health commodities availability in Tanzania. PLOS Glob Public Health. 2023;3:e0001867. 37155608 10.1371/journal.pgph.0001867 PMC10166559

[10] Joram F, Hiliza J, Nathanael S, Anaeli A. Implementation of direct health facility financing in the rural District of Kigoma in Western Tanzania. Pan Afr Med J. 2023;46:19. 10.11604/pamj.2023.46.19.41052. 10.11604/pamj.2023.46.19.41052 PMC10683170 38035157

[11] Kapologwe NA, Kibusi SM, Borghi J, Gwajima DO, Kalolo A. Assessing health system responsiveness in primary health care facilities in Tanzania. BMC Health Serv Res. 2020;20:104. 32041609 10.1186/s12913-020-4961-9 PMC7011252

[12] MOHCDGEC. The national roadmap strategic plan to improve reproductive, maternal, newborn, child & adolescent health in Tanzania (2016–2020). URT. https://www.globalfinancingfacility.org/sites/gff_new/files/Tanzania_One_Plan_II.pdf. Accessed 5 Nov 2023.

[13] Dominico S, Serbanescu F, Mwakatundu N, Kasanga MG, Chaote P, Subi L, et al. A comprehensive approach to improving emergency obstetric and newborn care in Kigoma, Tanzania. Glob Health Sci Pract. 2022;10(2):e2100485. 10.9745/GHSP-D-21-00485. 10.9745/GHSP-D-21-00485 PMC9053146 35487553

[14] Muganyizi P. Availability, coverage and geographical distribution of emergency obstetric and neonatal care services in tanzania mainland. JGO. 2017;5:1.

[15] Ueno E, Adegoke AA, Masenga G, Fimbo J, Msuya SE. Skilled birth attendants in Tanzania: a descriptive study of cadres and emergency obstetric care signal functions performed. Matern Child Health J. 2015;19:155–69. 24791974 10.1007/s10995-014-1506-z

[16] Hokororo J, Katembo A-G, Kinyenje E, Amani D, Ndjovu A, Eliakimu E, et al. Continuity of essential health services amidst COVID-19 pandemic in Tanzania: a pre and post implementation support assessment. Discov Health Syst. 2023;2:19.

[17] Kapologwe NA, Meara JG, Kengia JT, Sonda Y, Gwajima D, Alidina S, et al. Development and upgrading of public primary healthcare facilities with essential surgical services infrastructure: a strategy towards achieving universal health coverage in Tanzania. BMC Health Serv Res. 2020;20:218. 32183797 10.1186/s12913-020-5057-2 PMC7076948

[18] LaFond A, Cherney M. A theory of change for guiding the integration of human-centered design into Global Health Programming. Glob Health Sci Pract. 2021;9(Suppl 2):S209–16. 34845044 10.9745/GHSP-D-21-00334 PMC8628502

[19] Thaddeus S, Maine D. Too far to walk: maternal mortality in context. Soc Sci Med. 1994;38:1091–110. 8042057 10.1016/0277-9536(94)90226-7

[20] MOHSW. Health Sector Strategic Plan IV July 2015-June. 2020. URT. https://mitu.or.tz/wp-content/uploads/2021/07/Tanzania-Health-Sector-Strategic-Plan-V-17-06-2021-Final-signed.pdf. Accessed 13 Jun 2023.

[21] Clifford KL, Zaman MH. Engineering, global health, and inclusive innovation: focus on partnership, system strengthening, and local impact for SDGs. Glob Health Action. 2016;9:30175. 26790462 10.3402/gha.v9.30175 PMC4720687

[22] Coles E, Anderson J, Maxwell M, Harris FM, Gray NM, Milner G, et al. The influence of contextual factors on healthcare quality improvement initiatives: a realist review. Syst Rev. 2020;9:94. 32336290 10.1186/s13643-020-01344-3 PMC7184709

[23] UNDESA. The 17 Goals, Sustainable Development. UNDESA. https://sdgs.un.org/goals. Accessed 22 Oct 2023.

[24] Nyamtema AS, Mwakatundu N, Dominico S, Mohamed H, Pemba S, Rumanyika R, et al. Enhancing maternal and Perinatal Health in under-served remote areas in Sub-saharan Africa: a Tanzanian Model. PLoS ONE. 2016;11:e0151419. 26986725 10.1371/journal.pone.0151419 PMC4795747

[25] Nyamtema AS, Mtey G, LeBlanc JC. Requirements and costs for scaling up comprehensive emergency obstetric and neonatal care in health centres in Tanzania. Afr J Reprod Health. 2021;25(3s):84–91.37585862

[26] Nyamtema AS, LeBlanc JC, Mtey G, Tomblin Murphy G, Kweyamba E, Bulemela J, et al. Scale up and strengthening of comprehensive emergency obstetric and newborn care in Tanzania. PLoS ONE. 2022;17:e0271282. 35802730 10.1371/journal.pone.0271282 PMC9269945

[27] Otolorin E, Gomez P, Currie S, Thapa K, Dao B. Essential basic and emergency obstetric and newborn care: from education and training to service delivery and quality of care. Int J Gynaecol Obstet. 2015;130(Suppl 2):S46–53. 26115858 10.1016/j.ijgo.2015.03.007

[28] Bohren MA, Hunter EC, Munthe-Kaas HM, Souza JP, Vogel JP, Gülmezoglu AM. Facilitators and barriers to facility-based delivery in low- and middle-income countries: a qualitative evidence synthesis. Reprod Health. 2014;11:71. 25238684 10.1186/1742-4755-11-71 PMC4247708

[29] Kohi TW, Mselle LT, Dol J, Aston M. When, where and who? Accessing health facility delivery care from the perspective of women and men in Tanzania: a qualitative study. BMC Health Serv Res. 2018;18:564. 30021571 10.1186/s12913-018-3357-6 PMC6052684

[30] Mosley PD, Saruni K, Lenga B. Factors influencing adoption of facility-assisted delivery - a qualitative study of women and other stakeholders in a Maasai community in Ngorongoro District, Tanzania. BMC Pregnancy Childbirth. 2020;20:100. 32050919 10.1186/s12884-020-2728-2 PMC7014728

[31] Kalolo A, Kapologwe NA, Samky H, Kibusi SM. Acceptability of the Direct Health Facility Financing (DHFF) initiative in Tanzania: a mixed methods process evaluation of the moderating factors. Int J Health Plann Manage. 2022;37:1381–401. 34952982 10.1002/hpm.3402

[32] Kapologwe NA, Kalolo A, Kibusi SM, Chaula Z, Nswilla A, Teuscher T, et al. Understanding the implementation of Direct Health Facility Financing and its effect on health system performance in Tanzania: a non-controlled before and after mixed method study protocol. Health Res Policy Syst. 2019;17:11. 30700308 10.1186/s12961-018-0400-3 PMC6354343

[33] Tukay SM, Pasape L, Tani K, Manzi F. Evaluation of the direct health facility financing program in improving maternal health services in pangani district, tanzania. Int J Womens Health. 2021;13:1227–42. 34916854 10.2147/IJWH.S333900 PMC8669272

[34] Stigler GJ. The economies of scale. J Law Econ. 1958;1:54–71.

[35] Tsofa B, Molyneux S, Gilson L, Goodman C. How does decentralisation affect health sector planning and financial management? A case study of early effects of devolution in Kilifi County, Kenya. Int J Equity Health. 2017;16:151. 28911325 10.1186/s12939-017-0649-0 PMC5599897

[36] Kema KM, Komwihangiro J, Kimaro S. Integrated community based child survival, reproductive health and water and sanitation program in Mkuranga district, Tanzania: a replicable model of good practices in community based health care. Pan Afr Med J. 2012;13 Suppl 1:11. PMC3589253 23467915

[37] Joseph C, Maluka SO. The influence of community factors in the implementation of community-based interventions to improve antenatal care: a qualitative study based on the IMCHA programme in Tanzania. Reprod Health. 2021;18:188. 34551794 10.1186/s12978-021-01225-5 PMC8456547

[38] Petrucka P, Bassendowski S, Dietrich-Leurer M, Spence-Gress C, Athuman Z, Buza J. Maternal, newborn and child health needs, opportunities and preferred futures in Arusha and Ngorongoro: hearing women’s voices. BMC Res Notes. 2015;8:773. 26654627 10.1186/s13104-015-1776-6 PMC4676882

